# Coexistence of nodal marginal zone B-cell lymphoma and multiple myeloma: A case report and literature review

**DOI:** 10.1097/MD.0000000000029219

**Published:** 2022-05-06

**Authors:** Manzhi Wang, Yan Guo, Lin Dong, Kehong Bi

**Affiliations:** Department of Hematology, The First Affiliated Hospital of Shandong First Medical University & Shandong Provincial Qianfoshan Hospital, Jinan, Shandong, China.

**Keywords:** case report, multiple myeloma, nodal marginal zone B-cell lymphoma, prognosis, treatment

## Abstract

**Rationale:**

Nodal marginal zone B-cell lymphoma (NMZL) is a relatively rare indolent lymphoma that is the least common subtype of marginal zone B-cell lymphoma. Furthermore, coexistence of lymphoma and multiple myeloma (MM) is rare. Here, we report a case of the coexistence of NMZL and MM.

**Patient concerns:**

The patient was a 66-year-old man with pain in the left rib and lower left back. Enlarged lymph nodes were palpable in the cervical and inguinal regions, the biggest of which was 4.0 × 2.0 cm in size in the left groin.

**Diagnosis:**

Pathological and immunohistochemical findings of the left inguinal lymph node revealed NMZL. The patient met the diagnostic criteria for symptomatic MM. Therefore, the patient was diagnosed with NMZL and lambda-type MM.

**Intervention:**

We adopted a bortezomib, liposomal adriamycin, and dexamethasone (PAD) chemotherapy regimen mainly for MM.

**Outcomes:**

After 3 cycles of treatment, the patient achieved complete remission of myeloma. A 50% decrease in enlarged lymph node size was observed.

**Lesson:**

To our knowledge, this is the first case report of the simultaneous occurrence of NMZL and MM. Further studies are required to explain the association between these 2 tumors and improve treatment selection.

Nodal marginal zone B-cell lymphoma (NMZL) is a relatively rare indolent lymphoma, accounting for 1% to 2% of all lymphoid neoplasms, and represents the least common subtype of marginal zone B-cell lymphoma (approximately 10%).^[1]^ The median age at presentation is 50 to 67 years, and there is a female preponderance in some studies.^[2,3]^ NMZL tends to present with an advanced disease stage in >50% of cases. B-symptoms are observed in less than 15% of patients, and bone marrow involvement is apparent in 30% to 45% of cases.^[4]^

Multiple myeloma (MM) is a tumor that generally affects elderly individuals. It is a neoplastic plasma cell disorder that can be distinguished by the clonal proliferation of malignant plasma cells in the bone marrow and the presence of monoclonal proteins in the blood or urine.^[5]^ Eventually, numerous complications tend to appear alongside the disease, which are typically summarized by the acronym CRAB (hypercalcemia, renal failure, anemia, and bone lesions). In recent years, the overall survival and progression-free survival of MM have been increasing due to the application of targeted drugs, including bortezomib, thalidomide, and lenalidomide.^[6]^

The simultaneous appearance of lymphoma and MM is a rare phenomenon.^[7]^ The current study is the first to report the coexistence of NMZL and MM, and a good outcome was achieved using bortezomib-containing chemotherapy.

## 1 Case presentation

A 66-year-old man was admitted to the hospital in March 2021 because of pain in the left ribs and lower left back. The patient had a history of coronary atherosclerotic heart disease and cerebral infarction. Since the symptoms first occurred, the patient had experienced no night sweats or weight loss and had not received any relevant medical treatment.

Upon physical examination, the patient's temperature was 36.3°C (normal range, 36–37°C). We could touch the enlarged lymph nodes in the cervical and inguinal regions, and the biggest one was 4.0 × 2.0 cm in size in the left groin. The remaining physical examination results were negative.

The blood test results on admission revealed a white blood cell count of 7900/μL (normal range: 3400–9500/μL), a hemoglobin level of 12.2 g/dL (13.0–17.5 g/dL), a platelet count of 193,000 cells/μL (125,000–350,000 cells/μL), lactate dehydrogenase level of 208 U/L (135–225 U/L), serum albumin level of 40.4 g/L (40–55 g/L), and serum beta-2 microglobulin level of 1.98 mg/L. Lambda type monoclonal protein was detected using both serum and urine immunoelectrophoresis. In addition, the serum-free light chain κ/λ level (6.95 [3.30–19.40] mg/L/1220 [5.71–26.30] mg/L = 0.0057 [0.26–1.65]) and the urine-free light chain κ/λ level (23.80 [0.39–15.10] mg/L/1770 [0.81–10.10] mg/L = 0.0134 [0.461–4.00]) significantly decreased. Furthermore, the mutation of gene MYD88-L265P was negative.

**Figure 1:**
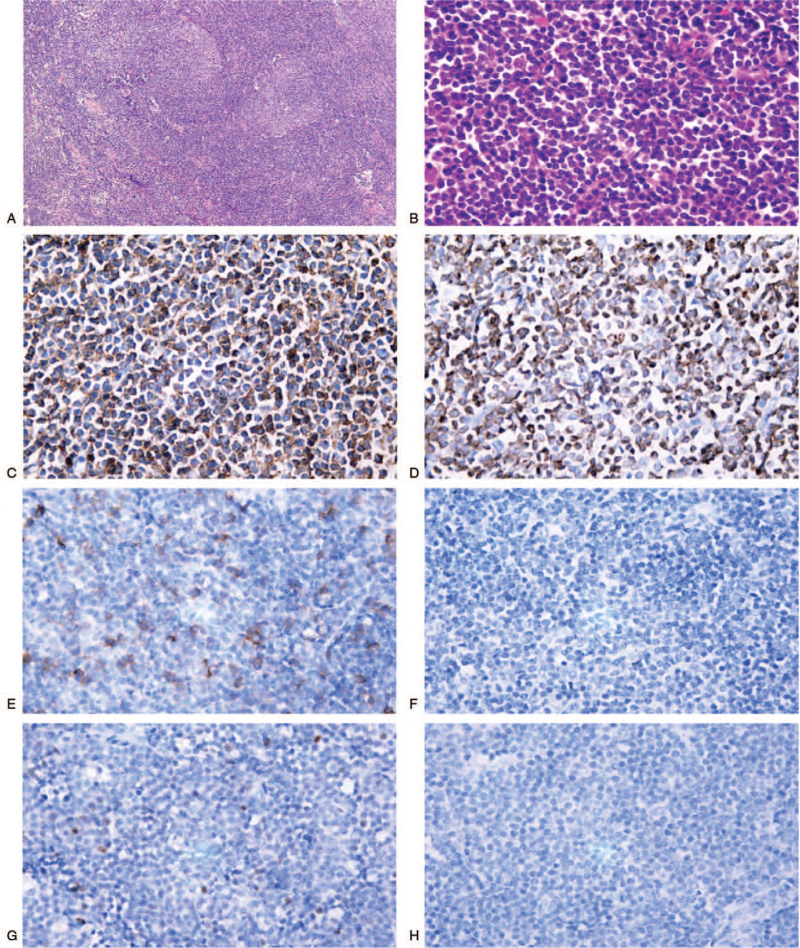
Biopsy of the enlarged lymph node of the left inguinal region. (A) A small-cell lymphoid proliferation surrounds reactive follicles and expands into the interfollicular areas. (Hematoxylin-Eosin staining at low magnification). (B) The neoplastic cells are composed of variable numbers of marginal zone (monocytoid) B cells and medium to large cells that are centroblast-like. (Hematoxylin-Eosin staining ×400). The tumor cells stained strongly positive for CD20 (C) and BCL2 (D), partially positive for CD5 (E), and almost negative for CD23 (F), bcl-6 (G), and CD138 (H).

The pathological finding of the left inguinal lymph node showed small-cell lymphoid proliferation surrounding the reactive follicles and expansion into the interfollicular areas. Neoplastic cells were composed of variable numbers of monocytic and centroblast-like cells. Immunohistochemistry revealed that the cells were positive for CD20 and Bcl-2 and negative for CD5, CD23, CD15, and CD138. The with a ki-67 level of 30% (Fig. 1). Finally, the patient was diagnosed with NMZL according to the 2016 World Health Organization classification.^[8]^

Osteolytic lesions in the lower cervical spine, upper thoracic, fifth posterior rib on the right, and eighth rib on the left were identified using computed tomography scan.

**Figure 2:**
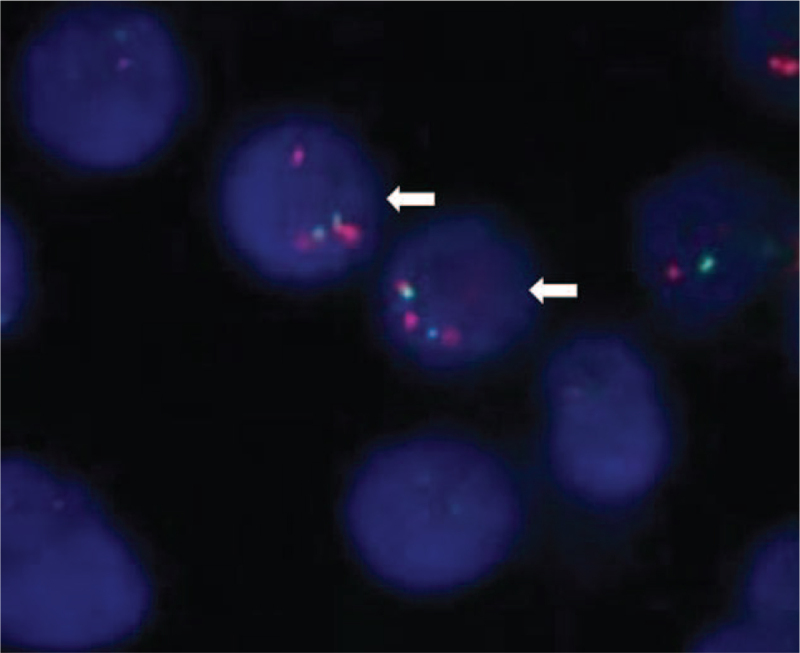
1q21 amplification by FISH analysis. CKS1B probe for 1q21-22 (orange signals), and CDKN2C probe for 1p32.3 (green signals). We detected 1q21 amplification (2 green signals, 3 orange signals) in >10% of tumor cells; white arrows indicate typical tumor cells with 1q21 amplification. FISH = fluorescence in situ hybridization.

Bone marrow cytomorphological examination revealed 10% immature plasma cells and 24.5% lymphoma cells. The cells were further confirmed as monoclonal cells by flow cytometry (9.86% monoclonal B cells: CD19+, CD20+, FMC7+, CD38+, CD200+, Kappa+, intracellular Kappa+, CD79b+, CD5+, CD22+; and 1.23% monoclonal plasma cells: CD38+, CD138+, Lambda+, CD56+). 1q21 gene amplification was detected using fluorescence in situ hybridization (FISH) analysis (Fig. 2).

The patient was diagnosed with lambda-type MM (International Staging System I, Durie and Salmon stage III) and NMZL (clinical stage IVA, IPI 2). Owing to the more indolent course of NMZL, we opted to perform watchful waiting. For MM, treatment by chemotherapy was initiated with PAD (bortezomib [1.3 mg/m^2^, days 1, 4, 8, 11] + liposomal adriamycin [40 mg, day 4] + dexamethasone [20 mg, days 1, 2, 4, 5, 8, 9, 11, 12]) in a 3 to 4 weeks cycle. The patient achieved complete remission after 3 cycles of PAD for myeloma. The bone pain disappeared. Lambda type monoclonal protein was not detected on both serum and urine immunoelectrophoretic tests. A 50% decrease in enlarged lymph node size was observed. The entire process of chemotherapy was completed in the hospital, so the intervention adherence was good. In addition, the patient had no serious adverse reactions, such as peripheral neuritis, cardiac complications, or infection. We found that he was not a candidate for autologous stem cell transplantation in view of his heart comorbidities and personal preference, and opted to continue treatment with bortezomib until progression or toxicity.

NMZL has a heterogeneous morphology in terms of both architecture and cytology.^[9]^ Different patterns of lymph node infiltration have been reported, including marginal zone like/perifollicular, inverse follicular, perisinusoidal, follicular via colonization of reactive follicles, and diffuse.^[2]^ Cytologically, NMZL shows varying proportions of small lymphocytes, marginal zone-like cells, centrocyte-like cells, and monocytoid B cells.^[10]^ In addition, NMZL shares common immunophenotypic markers, including CD19, CD20, and CD22; typically, they are negative for CD5, CD10, and CD23.^[11]^

In our patient, pathological findings showed a marginal zone-like pattern. Neoplastic cells were composed of monocytic and centroblast-like cells. Based on the typical immunohistochemical phenotype, the patient was diagnosed with NMZL. The patient also had symptoms of bone pain, which seemed unexplained by the NMZL. Therefore, we performed further examinations and found that the patient met the criteria for symptomatic MM.

There are many ways to investigate whether there is a clonal relationship between the 2 concomitant B-cell neoplasms, such as the analysis of immunoglobulin isotypes, light chain restrictions, idiotypes, and gene rearrangement.^[11]^ In this case, the 2 neoplastic groups had different restricted light chain expression. In conclusion, we believe that the patient had NMZL and MM.

B-cell lymphoma and MM rarely occur simultaneously or in succession. We reviewed similar cases from the PubMed database and found that the types of B-cell lymphoma coexisting with MM were Hodgkin's lymphoma,^[12,13]^ chronic lymphocytic leukemia/small lymphocytic lymphoma,^[14,15]^ diffuse large B-cell lymphoma,^[16]^ follicular lymphoma^[17]^ and mantle cell lymphoma.^[18]^ The most common type was chronic lymphocytic leukemia/small lymphocytic lymphoma. The possible pathogenetic mechanisms of the occurrence of both B-cell lymphoma and MM are as follows: 1 malignant disease may evolve from another. The development of high-grade malignant lymphoma in patients with chronic lymphocytic leukemia was first reported by Richter in 1928, and transformation to high-grade lymphoma is now a well-known event in other low-grade malignancies of the B-cell lineage.^[19]^ Additionally, malignant B-cell lymphoma clones may mature and give rise to MM. However, both malignancies can also be different manifestations of the original neoplastic clones.^[20]^

As the specific mechanisms underlying the simultaneous presentation of 2 B-cell malignancies have not yet been elucidated, there are no uniform treatment guidelines. For NMZL, in cases of low tumor burden, a watchful waiting strategy is usually employed, whereas in disseminated stage disease, immunochemotherapy (rituximab plus chemotherapy with or without anthracycline) is considered an appropriate option.^[10]^ For this patient, due to the more indolent course and low tumor burden of NMZL, we mainly treated MM. After all, we chose the treatment containing bortezomib with liposomal adriamycin and dexamethasone and finally achieved good results for both neoplasms.

In summary, for the first time, we reported a case of indolent NMZL coexisting with MM that enriched the categories of lymphomas appearing simultaneously with MM. Since the 2 neoplastic cells had different restricted light chain expressions, we suspect that this was an extremely rare case of nearly simultaneous presence of 2 independent NMZL and MM clones. The diagnosis and treatment process of this patient provided a reference for indolent B-cell lymphoma and MM. Finally, we also wish to study the mechanism of the occurrence of both lymphoma and MM and standardize the treatment for such patients.

## Acknowledgments

We wish to thank the patients participating in the study and all the caregivers and physicians in the Department of Hematology, The First Affiliated Hospital of Shandong First Medical University, and Shandong Provincial Qianfoshan Hospital. In addition, we gratefully acknowledge Guihui Zhang, Min Li, Hai Zhao, and other members of the Department of Pathology, the First Affiliated Hospital of Shandong First Medical University, and Shandong Provincial Qianfoshan Hospital for their invaluable assistance throughout the preparation of the original manuscript.

## Author contributions

MZW collected data. MZW and KHB analyzed the literature and wrote the manuscript. YG and LD reviewed and edited the manuscript. All authors have read and approved the final manuscript.

**Conceptualization:** Kehong Bi.

**Data curation:** Manzhi Wang.

**Funding acquisition:** Manzhi Wang.

**Investigation:** Kehong Bi, Lin Dong, Yan Guo.

**Writing – original draft:** Kehong Bi, Manzhi Wang.

**Writing – review & editing:** Lin Dong, Yan Guo.
